# A lifelong study of a pack Rhodesian ridgeback dogs reveals subclinical and clinical tick-borne *Anaplasma phagocytophilum* infections with possible reinfection or persistence

**DOI:** 10.1186/s13071-018-2806-8

**Published:** 2018-04-12

**Authors:** Emil Hovius, Arnout de Bruin, Leo Schouls, Joppe Hovius, Niels Dekker, Hein Sprong

**Affiliations:** 1 Amphipoda, Biology and Veterinary Science, Veldhoven, The Netherlands; 2 Center for Infectious Disease Control, National Institute for Public Health and the Environment, Bilthoven, The Netherlands; 3 Center for Experimental and Molecular Medicine, Academic Medical Center, University of Amsterdam, Amsterdam, The Netherlands; 4 Department of Infectious Diseases and Immunology, Veterinary Faculty, Utrecht University, Utrecht, The Netherlands

**Keywords:** *Anaplasma phagocytophilum*, Persistence, Re-infection, Rhodesian ridgeback dog, Fever, Thrombocytopenia, Canine granulocytic anaplasmosis

## Abstract

**Background:**

Various tick-borne infections often occur without specific clinical signs and are therefore notoriously hard to diagnose separately in veterinary practice. Longitudinal studies over multiple tick seasons performing clinical, serological and molecular investigations in parallel, may elucidate the relationship between infection and disease. In this regard, six related Rhodesian Ridgeback dogs living as a pack became subject of lifetime studies due to ongoing tick infestations and recurring clinical problems. Blood samples for diagnostic tests were obtained throughout the years 2000 to 2009.

**Methods:**

Data collected from clinical observations**,** hemograms, serology and detection of *Anaplasma phagocytophilum*, either by microscopy or by DNA amplification and typing, were placed in a time line. This dataset essentially presents as a prospective study enabling the association of the *Anaplasma* infections with occurring disease.

**Results:**

All six dogs were infected, and two of them developed particular clinical symptoms that could be associated with *Anaplasma* infections over time. More specifically, episodes of general malaise with fever and purpura with thrombocytopenia and bacterial inclusions in granulocytes, were found concurrently with *Anaplasma* DNA and specific antibodies in peripheral blood samples. DNA from *A. phagocytophilum* variant 4 (of *16S* rRNA) was found in multiple and sequential samples. DNA-sequences from variant 1 and the human granulocytic ehrlichiosis (HGE) agent were also detected.

**Conclusions:**

In this study two lifelong cases of canine anaplasmosis (CGA) are presented. The data show that dogs can be naturally infected concurrently with *A. phagocytophilum* variant 1, variant 4 and the HGE agent. The ongoing presence of specific antibodies and *Anaplasma* DNA in one dog indicates one year of persisting infection. Treatment with doxycycline during recurring clinical episodes in the other dog resulted in transient clinical improvement and subsequent disappearance of specific antibodies and DNA suggesting that re-infection occurred.

**Electronic supplementary material:**

The online version of this article (10.1186/s13071-018-2806-8) contains supplementary material, which is available to authorized users.

## Background

*Anaplasma phagocytophilum* one of the pathogens found in *Ixodes ricinus*, is capable of infecting a broad range of animal species [1, 2]. After introduction into the skin with tick saliva, the bacterium is incorporated in the vacuole of a neutrophilic granulocyte, where multiplication results in a microscopically visible morula and dysregulation of bactericidal neutrophilic functions [3]*.* Clinical signs have been described in sheep, cattle, horses, dogs and humans [2, 4, 5]. After taxonomic revision, the (sub)species infecting these animals were clustered in one species designated *A. phagocytophilum* but remain classed as variants [6]. These variants are identified on the basis of sequence differences in the *16S* rRNA or other genes and in dogs they appear to determine the clinical outcome [7–9]. Domesticated animals and man share their ecotype variants with red deer, swine and hedgehogs [10]. The European hedgehog (*Erinaceus europaeus*) can be regarded as one of the most important wildlife reservoirs for *A. phagocytophilum* maintaining the infection cycle to humans and canines [11–13].

Tick-borne infections in the dog, when symptomatic, do not result in a differentiable clinical picture [14]. No single symptom can be appointed as pathognomonic for an *A. phagocytophilum* infection, but fever with severe pain and thrombocytopenia warrants serological and molecular testing for *Anaplasma*. Other possible signs include pale mucous membranes with petechiae, scleral bleeding, tachypnoea, enlarged lymph nodes, gastrointestinal signs (tense abdomen/splenomegaly) and lameness and notably a reluctance to move [15, 16]. An active *Anaplasma*-infection can be diagnosed by microscopic detection of morulae in granulocytic white blood cells or by demonstrating emerging specific antibodies (seroconversion) [17]. Fever intensity and severity of thrombocytopenia relates to the number of infected neutrophils. Detection of *Anaplasma* DNA in peripheral blood is more sensitive presenting before and after morulae. Persistence of infection during two months or more is generally encountered in experimentally infected, deliberately immunosuppressed, dogs [14, 18–20]. However, persistence of infection in household dogs is debated and has not yet been determined unequivocally [15, 21].

Lifelong investigations of six frequent tick infested Rhodesian Ridgeback dogs comprising a 9-year period of parallel observations, on clinical signs and laboratory assays, reveals symptomatic anaplasmosis in two dogs.

## Methods

### Dogs and diagnostic sampling

**Fig. 1:**
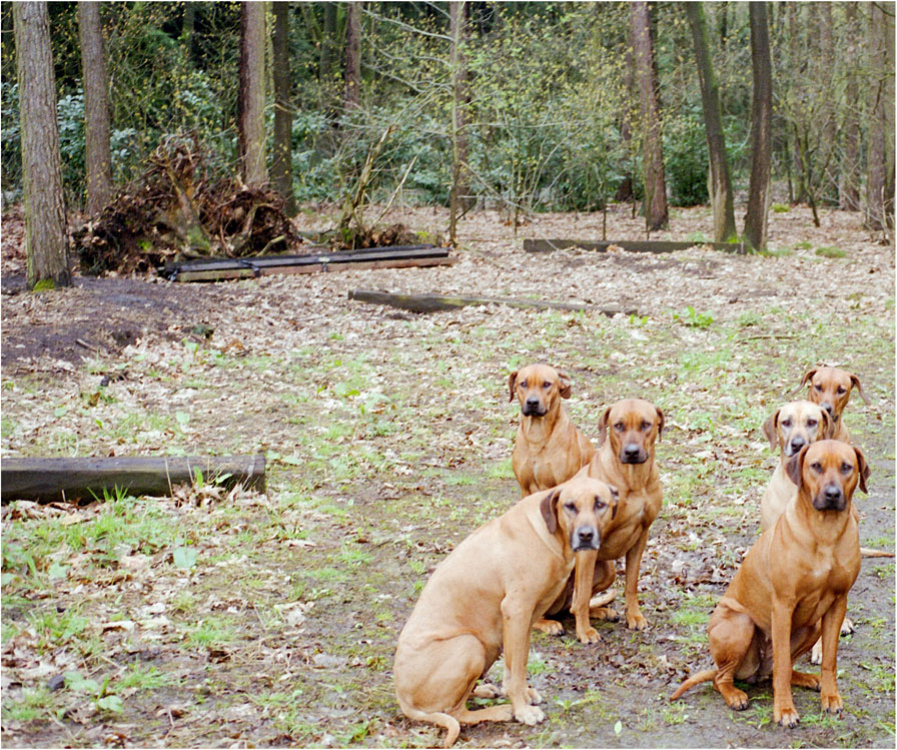
The pack, six female Rhodesian ridgeback dogs in the large garden adjacent to oak-pinewood forests

Six female Rhodesian ridgeback dogs living in one household were under care of a local veterinarian (KEH). The dogs home was situated at the village border (Veldhoven, Kempen area, province of Brabant, the Netherlands) with a large semi-natural garden including natural woodland. The dogs roamed loose and were daily walked in surrounding woodlands. Dog M12 (born 1994) was mother of dogs L11, L12, L13 (born 1998) and L21 (born 1999); both litters from one sire (Fig. 1). Dog N00 was distantly related and one year older than M12. Dogs were presented for consultation whenever health problems were suspected, and results of the clinical examinations were filed. Routine vaccinations were only administered in the first years. Tick repellents or acaricidal topicals with high efficacy were not available at the time of study, and thus were not applied. When diagnostically necessary venous blood samples were taken and examined in view of the clinical presentation. Ultimately, when severely ill and elder, the dogs were euthanized on the owner request, however one dog died naturally. With the owners’ informed consent, tissues for histopathology were subsequently obtained from all dogs except N00 and examined by a certified veterinary pathologist. In addition, a sample of spleen tissue was frozen for later use. Venous blood was sent to a reference laboratory for automated red and white blood cell counts. Blood smears were microscopically viewed in house for cell forms and inclusion bodies and in later years, cell differentiations and counts were also done in house. Blood chemical analysis was performed on all samples with an in house automatic analyzer (Spotchem, Menarini, Valkenswaard, the Netherlands). Routinely, urea, creatinine, glucose, total protein, albumin, alkaline phosphatase, glutamate transaminase and total calcium were tested.

### Serological methods

*Anaplasma*-specific serology was performed at different laboratories and with in-house test kits. Immunofluorescent antibody tests for at the time named *Ehrlichia phagocytophila* and *Ehrlichia canis* were performed on the 2001 samples. An immunoblot with the p44 protein of *A. phagocytophilum*, was performed on the 2001 through 2003 samples. Later in 2009, the SNAP® 4Dx-test (IDEXX Laboratories, Westbrook, ME, USA) that also detects antibodies against the p44 protein became available and all sera were retrospectively tested in house. This in-house test also detects antibodies against *E. canis*, the C6 antigen of *Borrelia burgdorferi* (*sensu lato*) and heartworm antigens. Moreover, a whole cell Elisa for the detection of *B. burgdorferi* (*s*.*l*.) antibodies was used on selected sera.

### Molecular agent detection

For detection of *Anaplasma* DNA, blood samples were distributed over several laboratories using different targets. DNA extraction was performed as described for *Anaplasma* studies in Swedish dogs [22]. Importantly, *16S* rRNA gene-based PCR assay was performed in which the DNA products were subjected to a reversed line blot (RLB) detecting the different variants of *A. phagocytophilum* [23, 24]. Briefly, oligonucleotide probes characterizing the *A. phagocytophilum* variants were covalently coupled in a linear manner to slots of a miniblotter (Immunetics, Cambridge, Mass, USA). The biotin labeled denatured PCR product was applied into the slots to hybridize with the probes. Hybridization was confirmed by visualizing the chemiluminescence reaction after coupling of biotin and streptavidin-peroxidase. Probes employed were specific for *A. phagocytophilum* variants 1 and 2 mostly found in cattle and sheep, and variants 3 and 4 found in ticks and dogs, for the variant most found in horses *Ehrlichia equi* named “*E*. *equi*”, and for the variant first detected in a human patient; “human granulocytic ehrlichiosis”, abbreviated as “HGE agent” (now the agent of human granulocytic anaplasmosis, HGA agent). GenBank accession numbers of these variants are: *A. phagocytophilum* variant 1, M73220, formerly *Ehrlichia phagocytophila* Old Sourhope, from sheep from Scotland [25]; *A. phagocytophilum* variant 2, AF336220, formerly new variant from sheep Norway [24]; *A. phagocytophilum* variant 3, AJ242784, formerly Swedish Tick *Ehrlichia* type IIb [26]; *A. phagocytophilum* variant 4, AJ242783, formerly Swedish Tick *Ehrlichia* type Ib and Rosa dog isolate [22]; *E*. *equi*, AF036646; HGE agent, U02521, Human Granulocytic *Ehrlichia*, from man Wisconsin USA, [27].

In 2014, frozen spleen samples of 5 of the 6 dogs and selected known positive full blood samples of dog M12 and L11, were tested by PCR. DNA was extracted using the Qiagen DNeasy® Blood & Tissue Kit (Qiagen, Venlo, the Netherlands) according to the manufacturer’s protocol for the purification of total DNA from blood and tissues. Detection of *A. phagocytophilum* and “*Candidatus* Neoehrlichia mikurensis” DNA by qPCR, was performed as described earlier [9, 28]. Briefly, for detection of DNA of both pathogens, a single multiplex qPCR assay was used, which targets specific regions of gene *msp2* (*p44,* Major Surface Protein 2) for *A. phagocytophilum*, and *groEL* (heat shock protein) for “*Candidatus* N. mikurensis”. Confirmation was obtained by conventional PCR using EphplgroEL-A.phago-F (5'-ATG GTA TGC AGT TTG ATC GC-3') and EphgroEL-A.phago-R (5'-TTG AGT ACA GCA ACA CCA CCG GAA-3') as primers with the following cycling program: 15 min at 95 °C, 40 cycles each consisting of 30 s at 94 °C, 57 °C and 72 °C, and final extension for 10 min at 72 °C.

## Results

Results of the molecular and serological tests are shown in Table 1. *Anaplasma phagocytophilum* DNA was detected in blood samples from two dogs; dog M12 and dog L11 (a daughter from the first litter of M12) with uniform results for all methods. In dog L11, *A. phagocytophilum* DNA was also detected in spleen tissue. Because the dogs were presented mainly whenever health problems occurred, the frequency and time of their tests was irregular (Additional file 1: Table S1). All six dogs were found serologically positive at least once in their lifetime.

**Table 1:** Results of serological and molecular diagnostic tests for anaplasmosis on the six Rhodesian ridgeback dogs performed at multiple time points throughout their lives

Clinic			PCR					Serology			
Dog (ID)	Birth- Death	Lethality	VMDC	RIVM 2001	RIVM 2003	RIVM 2014	Total	IFT	Immunoblot	Snap test	Total
			*16S* rRNA	*16S* rRNA	*16S* rRNA	*msp2* & *groEL*		Whole cell	P44	P44	
M12	1994–2001	Splenitis, fever and pain		1/1		1/3, spleen neg	2/3	1/2	2/2	0/4	2/5
L11	1998–2009	Malignant tumor in spleen, lung	1/4	2/3	2/9	3/4, spleen pos	5/16	2/3	3/12	3/18	7/21
L12	1998–2005	Splenitis, fever and pain		0/1	0/1	0/1, spleen neg	0/4	nd	0/3	3/5	3/7
L13	1998–2009	Haemangiosarcoma		0/1	0/1	0/2, spleen neg	0/5	nd	0/2	1/6	1/6
L21	1999–2005	Lymphoma		0/1	0/1	0/1, spleen neg	0/3	nd	1/2	0/6	1/7
N00	1995–2009	Metastasis mammae tumor		0/1	0/1	0/1, spleen nd	0/3	nd	1/1	2/6	3/6

*Notes*: Different laboratories were involved during the years, employing varying serological and molecular tests. Results are presented separately and overall: (i) The Veterinary Microbiological Diagnostic Center, Veterinary Faculty, Utrecht University, The Netherlands (VMDC) performed serological and molecular tests for ehrlichiae and *Babesia* as of 2001. Immunofluorescent tests for *E. phagocytophilum* and *E. canis* were performed on the 2001 samples; (ii) The National Institute of Public Health and Environment (RIVM) employed an immunoblot with the p44 protein of *A. phagocytophilum* and amplified the *16S* rRNA gene and confirmed the result with RLB (see Methods); (iii) The RIVM followed the same methods in 2003 with another team of technicians; (iv) The molecular detection by the RIVM in 2014 targeted a different set of genes (than in 2 and 3) in blood and spleen tissues (see Methods). The in-house SNAP® 4 Dx test (IDEXX) as serological assay was also employed.

*Abbreviations*: *neg*, negative, *nd* not determined, *pos* positive

Dog M12 was presented with a first episode of fever (40.0 °C) after her third litter; was painful and had atrophy of the right temporal muscle. The abdomen was bulgy and tense with a grossly enlarged spleen. Endometritis and enteritis was suspected and after ovariohysterectomy (OVH) and treatment with amoxicillin (10 mg bid for 3 days and one injection dexamethasone (10 mg) the dog recovered except for the atrophic right temporal muscle. A few months later she was put on non-steroid anti-inflammatory drugs (Metacam®, NSAID, Boehringer Ingelheim Vetmedica, Inc.) because of reluctance in lying down and getting up and the development of a “fer de lance” (an atrophied muscle segment) of the left loin muscle. The dog recovered completely, with the temporal muscle regaining normal proportions. However, although apparently healthy, she still had antibodies against *A*. *phagocytophilum* and *Anaplasma* DNA (*A*. *phagocytophilum* variant 4) in her blood. The blood smear and cell counts were abnormal and granulocytic morulae were observed (Additional file 1: Table S1**)**. Later, she was presented with a second fever episode (39.9–40.4 °C) with thrombocytopenia, anemia and intestinal bleeding (melena) apparently commencing with swelling of the left mandibular lymph node. Fever and melena resided after doxycycline (Ronaxan®, Merial, Lyon, France) therapy, but as the dog developed extensive subcutaneous exudate and appeared severely painful she was euthanized on the owners’ request. Blood chemistry for liver and kidney function had always been within the normal range. In all dogs, blood chemistry values were determined in parallel with all cell counts and all results were within the normal range (data not shown). No tumors were detected on complete gross pathology of dog M12 nor seen histologically in liver or spleen. The liver was congested and spleen infarctions were noted.

**Fig. 2:**
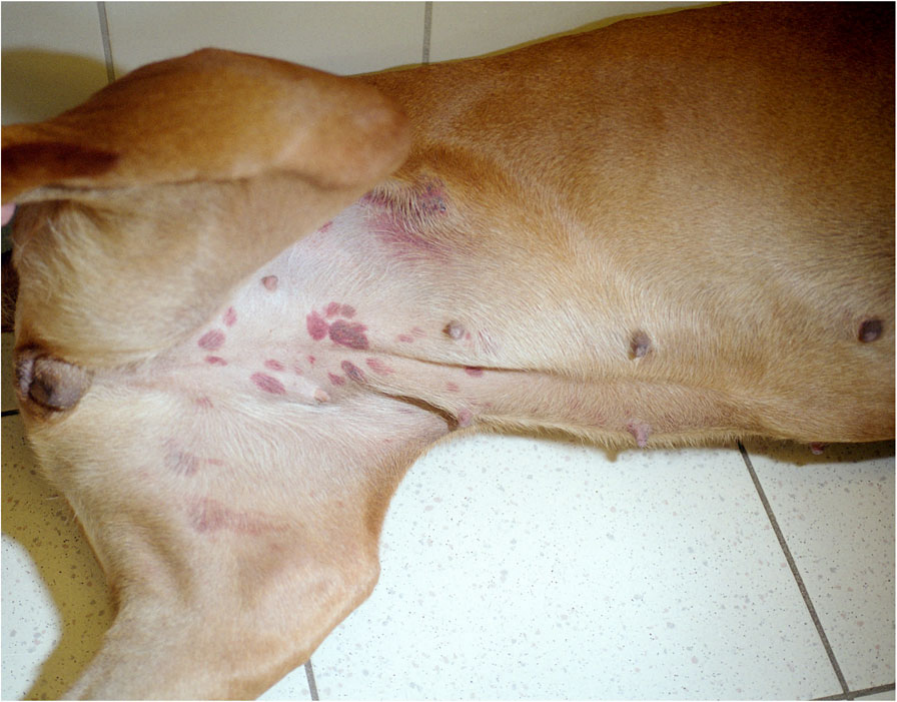
Dog L11 at first presentation in the veterinary clinic, with acute ecchymosis

**Fig. 3:**
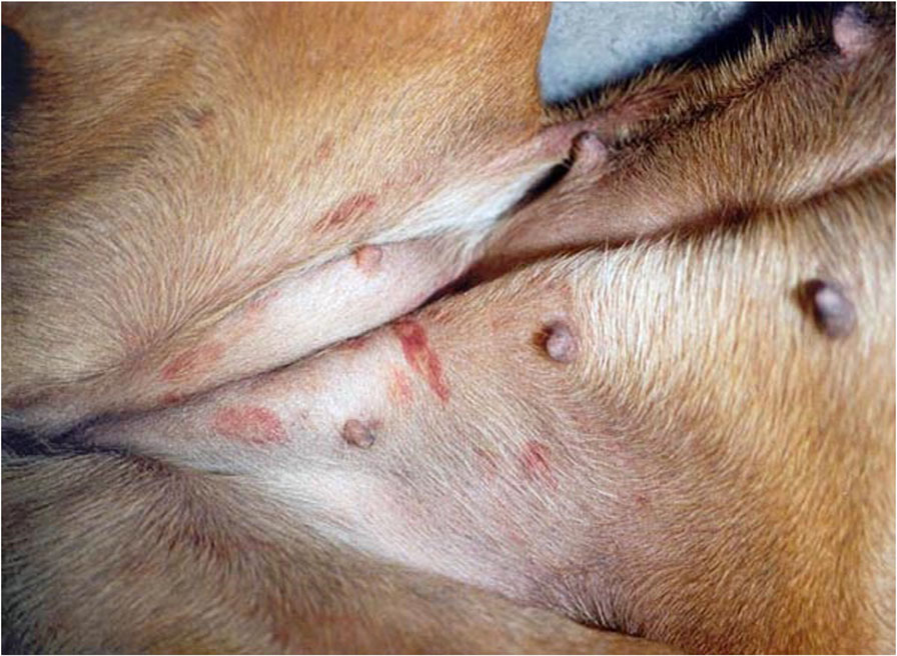
Dog L11 with the purpura residing within 5 days

Dog L11 and its littermates (L12, L13) had juvenile pyoderma when a few weeks old. This dog was provisionally diagnosed with four clinical episodes of canine granulocytic anaplasmosis (CGA) (see Additional file 1: Table S1**)**. In the first episode, doxycycline (Ronaxan®) therapy 5 mg per kilo bid for 10 days, was prescribed, in the later prescriptions the dose was 10 mg per kilo bid. During the first episode of disease, at the age of 3 years, dog L11 was presented with ecchymosis over the ventral abdominal skin (Fig. 2), and bleedings under the tongue, buccal mucosa as well on the inside of the ear. The purpura resided within 5 days (Fig. 3). No fever was noted. Thrombocytes were low (9 cells/nl) but recovered slightly within a week and remained at subnormal levels during the following 4 months. Neutrophilia was followed by lymphocytosis (Tables 2 and 3). DNA of *A. phagocytophilum* variants 1 and 4 was detected in blood. Doxycycline therapy was installed and 3 weeks later *Anaplasma* DNA was no longer detected; this therapy was repeated 3 more times. About 15 months later the dog developed a second episode of fever (39.6 °C) with thrombocytopenia (50 cells/nl) and morulae in neutrophils. Again, DNA of *A. phagocytophilum* variants 1 and 4 was found in blood samples and this time also the HGE agent was detected as well. Doxycycline therapy was again initiated. When tested three weeks later no *Anaplasma* DNA was detected. Antibodies disappeared from the circulation within 2 months. A third and fourth episode including fever thrombocytopenia and purpura with circulating antibodies occurred in 2005 and 2008. During the last episode, a slow growing lung tumor was detected. Fifteen months later the dog was euthanized. Her spleen tissue contained *A. phagocytophilum* DNA and histology revealed a severe splenitis with follicular hyperplasia and as in lung tissues contained undefinable tumor like cells. The other four dogs were also infected but no symptoms indicating possible CGA were observed (Additional file 1: Table S1**)**. One of these dogs, L12, was spayed (ovariohysterectomy) when she had a high fever (39.8 °C), supposedly caused by an endometritis. The laparoscopy showed a slightly enlarged uterus and a grossly enlarged spleen, histologically diagnosed as a splenitis which was negative for *Anaplasma* DNA. Morulae were observed in neutrophils, but blood samples were negative for *Anaplasma* DNA. At the moment SNAP® 4Dx test was positive for p44 antibodies. Dogs L13, L21 and N00 developed haemangiosarcoma, lymphoma and melanoma, respectively and mammary tumors as well. No morulae were observed, nor was DNA detected in peripheral blood or in the spleen. Anti-p44 antibodies were detected in all three dogs at least once during the live time. The SNAP® 4Dx test never detected specific antibodies against other tick-borne infections during this study (for dog L11 see Tables 2 and 3).

**Table 2:** Clinical episodes of dog L11 in 2001 and 2002 as determined by symptoms, hematology, serology and pathogen detection by PCR or microscopy. Four clinical episodes were detected in the years 2001, 2002, 2005 and 2008. Symptoms were not always accompanied by fever and bleeding tendency was not always apparent. Other symptoms as lymphadenopathy, muscle pain, lameness, reluctance to move and lethargy were also observed

Year		2001					2002						
Day/Month		23/04	27/04	14/05	29/06	28/08	09/02	07/03	21/06	03/09	23/09	25/09	18/10
Hematology (reference)													
Erythrocytes (> 5.5/pl)		7.05	6.21	6.78	7.84	7.56	7.11		6.75	7.15	5.72	6.33	6.46
Hemoglobin (> 8.8 mmol/l)		10.66	9.42	10.23	11.90	10.97	10.60		10.66	11.04	**8.37**	9.05	9.61
HT (> 42%)		51	46	48	61	56	54		49	52	**39**	43	50
MCV (65.5–75.5 fl)		71.70	73.60	71.10	**77.30**	73.90	75.40		73.20	72.50	67.80	68.20	**78.10**
MCH (0.90–1.55 pmol)		1.46	1.47	1.46	1.47	1.40	1.45		1.53	1.49	1.42	1.39	1.45
MCHC (20.9–22.3 mmol/l)		21.08	20.58	21.2	**19.59**	**19.59**	**19.78**		21.58	21.20	21.64	21.02	**19.10**
Thrombocytes (150–400/nl)		**9**	**50**	**131**	**23**	**17**	192		216	180	**50**	187	206
Leukocytes (5.9–13.8/nl)		11.4	11.6	**14.5**	8.4	8.9	7.9		7.9	8.2	7.8	6.8	8.5
Neutrophils (55–75%)		73	**79**	71	55	67	65		66	61	**78**	**42**	73
Lymphocytes (13–30%)		16	18	16	**35**	22	27		23	33	9	**52**	21
Monocytes (< 5%)		**8**	1	4	**9**	5	**6**		2	**5**	**9**	4	5
Eosinophils (< 4%)		2	1	**6**	1	**4**	1		**9**	1	0	2	1
Basophils (< 1%)		0		0	0	0	0		0	0	0	0	0
Staphylococcus (< 4%)		1	1	3	0	2	1		0	0	4	0	0
Serology													
*Anaplasma*	Immunoblot	neg^2^	neg^2^	neg^2^	neg^3^	neg^3^	neg^3^		neg^3^	neg^3^	**pos** ^3^	**pos** ^3^	**pos** ^3^
	IFT	**pos** ^1^	**pos** ^1^	neg^1^									
	Snap test		neg	neg	neg	neg			neg	neg		**pos**	neg
*Borrelia*	WHC-titer		80	40									
	C6 Snap test	neg	neg	neg	neg	neg			neg	neg		neg	neg
Coagulation test													normal
Microscopy (morulae)		**pos**	neg	neg	neg	neg	neg			neg	**pos**	neg	neg
PCR - DNA		**pos** ^2,4^	neg^2^	**pos** ^2^	neg^2^	neg^3^	neg^3^		neg^3^	neg^3,4^	**pos** ^1,3,4^	**pos** ^3^	neg^1,3^
RLB	A. phago variant 1	**pos** ^2^		neg							**pos** ^3^	neg	
	A. phago variant 2	neg		neg							neg	neg	
	A. phago variant 3	neg		neg							neg	neg	
	A. phago variant 4	neg		**pos** ^2^							**pos** ^3^	neg	
	*E. equi*	neg		neg									
	HGE agent	neg		neg							**pos** ^3^	**pos** ^3^	
Clinics													
	Body temperature	38.8	38.4	38.4	38.5	38.2	38.3	38.3	38.2		**39.6**		38.5
	Bleeding	**ecchm**	**resolv**	no	no	no	no	no	no	no	no	no	no
	Other	listless, lymphaden. R popliteus		pseudolac			lame	myositis, lays, pseudolac, lameness		pseudolac		dyspneu	cough
	Therapy		doxy			doxy		doxy	doxy			doxy	amox/clv
	Ticks on dog	yes					yes		yes	yes	yes		yes

*Notes:* Deviant or positive laboratory results are shown in bold. Serology and *Anaplasma* DNA detection employed different methods which were performed in different years and laboratories indicated by numbers 1 to 4. Assay method depended on laboratory and year of processing and is indicated as:^1^VMDC till 2008; ^2^RIVM in 2001; ^3^RIVM in 2003; and ^4^RIVM in 2014. The in-house SNAP® 4Dx test (IDEXX) was employed to determine antibodies against the p44 antigen, which was also detected by immunoblot in the first 2 years as was the IFA (immunofluorescent assay) in the first year. The agent detection by *16S* rRNA gene amplification and confirmed by reverse line blot hybridization (RLB) detecting variants of *A. phagocytophilum* was performed in the first two years. Time of doxycycline therapy and of tick collections from skin are notated

*Abbreviations*: *pos* positive, *neg* negative, *pseudolac* pseudo-lactation after estrous, *OVH* spaying by ovariohysterectomym *lymphaden* lymphadenopathy, *ecchm* ecchymosis, *resolv* resolving, *A. phago A. phagocytophilum*, *E. equi Ehrlichia equi*, *doxy* doxycyclin, *amox/clv* amoxicillin with clavulanic acid

**Table 3:** Clinical episodes of dog L11 from 2003 to 2009 as determined by symptoms, hematology, serology and pathogen detection by PCR or microscopy. Symptoms were not always accompanied by fever and bleeding tendency was not always apparent. Other symptoms as lymphadenopathy, muscle pain, lameness, reluctance to move and lethargy were also observed

Year		2003		2004		2005		2007		2008		2009
Day/Month		18/08	09/10	19/04	29/04	09/03	29/11	27/09	17/10	22/02	11/09	12/05
Hematology (reference)												
Erythrocytes (> 5.5/pl)			acanthosis	7.5		7.5	acanthosis	normal		normal	normal	
Hemoglobin (> 8.8 mmol/l)				11.4		11,4						
HT (> 42%)				52	**80**	**57**		**60**		50		
MCV (65.5–75.5 fl)				69.5								
MCH (0.90–1.55 pmol)				1.51								
MCHC (20.9–22.3 mmol/l)				21.8								
Thrombocytes (150–400/nl)		**>>>**	**<**	201		236	**<**	>>>		<<<	<	
Leukocytes (5.9–13.8/nl)		**16**	7	6.9		**14.5**	6	10		**30**	**60**	
Neutrophils (55–75%)		**87**	60	69		**80**	**83**	58		**80**	**85**	
Lymphocytes (13–30%)		9	25	23		7	11	**36**		**15**	**10**	
Monocytes (< 5%)		4	**12**	4		**13**		4		**4**	**5**	
Eosinophils (< 4%)		1	3	4		0	1	2		1		
Basophils (< 1%)				0		0	4	0				
Staphylococcus (< 4%)				0		0		0				
Serology												
*Anaplasma*	Immunoblot		neg									
	IFT											
	Snap test	neg	neg	neg	neg	neg	**pos**	neg	neg	**pos**	neg	
*Borrelia*	WHC-titer						80					
	C6 Snap test	neg	neg	neg	neg	neg	neg	neg	neg	neg	neg	
Coagulation/other tests			acanthosis			RF neg	Coombs +	RF neg				hypergamma proteinaemia
Microscopy (morulae)		neg	neg		neg	neg	neg	neg		**pos**	neg	
PCR - DNA		neg^3^	neg^1,3^			neg^1^				neg^1^		**spleen pos** ^4^
RLB	A. phago variant 1											
	A. phago variant 2											
	A. phago variant 3											
	A. phago variant 4											
	*E. equi*											
	HGE agent											
Clinics												
	Body temperature	38.6		38.9	**39.6**	**39.3**		38.9		**39.4**		
	Bleeding	no	no	no	no	no	**petechiae**	no	no	**erythema** belly		
	Other			Pseudolac	heart after OVH	non-healing wound		arthrosis in shoulders		dyspneu	lick granuloma	euthanasia
	Therapy				doxy	doxy					pleiomorphic malignant lung tumour	
	Ticks on dog		yes		yes		yes	yes				

*Notes:* Deviant or positive laboratory results are shown in bold. Serology and *Anaplasma* DNA detection employed different methods which were performed in different years and laboratories indicated by numbers 1 to 4. Assay method depended on laboratory and year of processing and is indicated as:^1^VMDC till 2008; ^2^RIVM in 2001; ^3^RIVM in 2003; and ^4^RIVM in 2014. The in-house SNAP® 4Dx test (IDEXX) was employed to determine antibodies against the p44 antigen, which was also detected by immunoblot in the first 2 years as was the IFA (immunofluorescent assay) in the first year. The agent detection by *16S* rRNA gene amplification and confirmed by reverse line blot hybridization (RLB) detecting variants of *A. phagocytophilum* was performed in the first two years. Time of doxycycline therapy and of tick collections from skin are notated

*Abbreviations*: *pos* positive, *neg* negative, *pseudolac* pseudo-lactation after estrous, *OVH* spaying by ovariohysterectomy, *lymphaden* lymphadenopathy, *ecchm* ecchymosis, *resolv* resolving, *A. phago A. phagocytophilum*, *E. equi* Ehrlichia equi, *doxy* doxycyclin

## Discussion

This longitudinal study, in essence a prospective study, shows that each member of a pack of six Rhodesian ridgeback dogs was infected at least once in its life with the tick-borne bacterium *A. phagocytophilum* (Additional file 1: Table S1). During the tick season, the dogs were almost daily infested with multiple ticks (see Tables 2 and 3 for dog L11) of which, in a previous study (2006–2009), at least 2.5% appeared to harbour *A. phagocytophilum* [29]. Canine granulocytic anaplasmosis (CGA) had not been diagnosed in the region before, however, establishing this diagnosis greatly depends on the timing of diagnostic sampling and its frequency. Experimental and clinical studies show that periods in which morulae and/or specific DNA are detectable in blood are relatively short; testing one sample may not be enough to exclude infection. Therefore, sequential sampling, as with dog L11, considerably increases the chance of detecting the agents, presence or short-lived antibodies [30]. Moreover, *A. phagocytophilum* infections vary in their clinical expression and the induced pathology is far less pronounced than that of other ehrlichiae [14].

Two of the six dogs in the pack (dog M12 and dog L11) showed symptoms as fever, bleeding tendencies, general painfulness (resulting in a reluctance to move) combined with thrombocytopenia and neutrophilia followed by lymphocytosis. These observations are similar to the first clinical and experimental case reports of *A. phagocytophilum* infections from Sweden [16, 17, 20] and are in line with the observations from clinical studies from other countries [3, 15, 31, 32]. Diagnosing CGA should be based on the synchronous occurrence of: (i) attributable symptoms, with (ii) abnormal findings in the blood smear including thrombocytopenia, and (iii) detection of active infection by molecular assays or microscopic observation of granulocytic morulae combined with specific antibody detection [15]. During its lifetime dog L11 showed these three criteria were synchronously at 4-time points (out of 17-time points that tested the three classes of observations simultaneously). This case can, therefore, be considered as an established case of CGA (Tables 2 and 3).

The in-house test measuring the specific p44 antibodies has a high sensitivity [33]. The *msp2/p44* gene consists of a large potential of pseudogenes enabling direct insertion into the expression site, inducing variability of the epitopes [34]. In a canine model, antibodies against the p44 antigen were shown to occur only for a short time after infection or to persist during continued subclinical or chronic infection [14, 18]. In naturally infected lambs, successive febrile peaks occur, and the p44 antibodies against the recombined epitopes appear with each remission whereas *Anaplasma* bacteria remain detectable for one day only [24, 30]. A close observation on the sequential measurements of dog L11 clarifies that as in lambs the antibody reaction against p44 was only measured for a short time period. Measuring anti-p44 antibodies thus appears as sensitive as agent detection and might indicate active and not past infection (Tables 2 and 3).

Dog M12 had two fever episodes within a year and was also sampled in between the two episodes. Initially, IFA antibodies were demonstrated during the first episode. Later, when samples taken between the two episodes, when the dog appeared clinically healthy, p44 antibodies, morulae and *Anaplasma* DNA were found (Additional file 1: Table S1). The dog was at first treated with a corticosteroid and a NSAID, because of supposed myositis, and with amoxicillin. During the second fever episode, when *Anaplasma* DNA was again detected, treatment with doxycycline was started. In retrospect, the aspects of this second fever episode, including bleeding tendencies (melena) together with thrombocytopenia, warranted the diagnosis CGA. However, the aberrant spleen, that was excised after euthanasia, at three weeks after the last positive blood sample, did not contain *Anaplasma* DNA, supposedly because of the doxycycline administered (3 weeks before). It is unclear whether the circulating *Anaplasma* bacteria detected in-between the two symptomatic periods were due to persistence of the agent or to reinfection. Persistence is a possible option as doxycycline had not yet been administered. However, reinfection is also possible since the ‘tick season’ had just begun. Notably, agent persistence has not been unequivocally demonstrated outside experimental settings [19, 21, 30].

Dog L12 was clearly infected with *A. phagocytophilum* in 2003, when morulae and antibodies were present. However, in 2004, when the dog fell ill showing symptoms such as fever, pain, an enlarged spleen and antibodies, these symptoms cannot be unequivocally attributed to the *Anaplasma* infection. Dogs L13, L21 and N00 showed symptoms indicating immunosuppression (tumor, worm infections, eosinophilia) at the time they tested positive for *A. phagocytophilum* antibodies but no CGA appropriate clinical signs nor thrombocytopenia were observed.

Exclusion of a causative role of other tick-borne agents for the observed symptoms was obtained by the SNAP® 4Dx test that did not reveal any sample positive for antibody responses to *B. burgdorferi* (*s*.*l*.) or *E. canis*. The latter, could, in the acute phase, also elicit bleeding and thrombocytopenia, and would present with morulae in monocytes, which were not detected. Moreover, the IFA for *E. canis*, employed in episode I of dog L11 was negative (data not shown). There were no inclusion bodies in thrombocytes that would indicate an *Anaplasma platys* infection and this bacterium has not been detected in Dutch ticks. Autochthonous canine babesiosis is excluded by specific PCR on the samples tested at the VMDC. Moreover, no piroplasms were observed in erythrocytes. The qPCR performed in 2014 was also able to detect DNA of “*Candidatus* N. mikurensis” but did not yield an amplification product for this target in the samples from this study. This novel agent was detected in 5% of questing ticks of the studied Kempen region and may cause symptoms similar to granulocytic anaplasmosis in dogs [28, 35, 36]. Low *Borrelia* whole cell values and absence of anti-*Borrelia* C6 antibodies in all six Ridgebacks is noteworthy. In the regional population of tick infested dogs, *Borrelia* infection rates of 100% have been found and canine Lyme borreliosis may be diagnosed [37–39].

Analysis of the *Anaplasma* PCR products using the reversed line blot (RLB) yielded three *A. phagocytophilum* variants. *Anaplasma phagocytophilum* variant 1 and variant 4 were found in episode I and II of dog L11. Remarkably, in the same quarter year, *A. phagocytophilum* variant 4 was also found in dog M12 (Additional file 1: Table S1). The third variant, “HGE agent” (HGA agent), was found during episode II of dog L11. The episode I infection is supposedly cleared by the doxycycline repeatedly applied, as *A. phagocytophilum* is very sensitive to this antibiotic [40]. Since antibodies were not detectable between episode I and II, reinfection (with the different variants not necessarily at the same time) would be the most parsimonious explanation. Re-infections have been demonstrated for naturally infected horses and humans, but so far not in naturally or experimentally infected dogs [15, 18, 21]. Persistence, on the other hand, with declining p44 antibody reactions has been described for naturally and experimentally infected lambs and experimentally infected dogs [20, 30, 41]. The dog could have remained infected with one or both of the two *A. phagocytophilum* variants, i.e. 1 and 4. A hesitantly recovering thrombocytopenia, as between episodes I and II, has been suggested to be an indication of non-circulating inactive bacteria at sub-detection levels [18]. Further, detailed characterization of the infecting strains in episodes I and II may reveal if persistence or reinfection has occurred.

The simultaneous detection of variants in one and the same sample of lambs is not a rule but rather an exception, indicating that “it may be difficult to obtain all variants involved in naturally infected animals” as stated by Stuen and colleagues [41]. Thus, according to Stuen et al. [41], although present the variants do not circulate together, implying that the results of samples different variants two days apart may nevertheless be interpreted as co-occurring. Thus, in dog L11 co-infection of two and three variants might have occurred in episodes I and II, respectively.

The pathogenic role of *A. phagocytophilum* variant 1 in the canine is uncertain; in the present study this classic sheep variant is reported in dogs for the first time. This variant may be an innocent bystander or alternatively divert the immune system, enabling the other variants to thrive. Previous studies show that the “HGE agent” (HGA agent) appears to be more pathogenic for dogs than other variants [7]. In addition, in nearly all case reports of CGA the causative agent is thought to be the “HGE agent” (HGA agent) based on 16S rRNA identities [7, 22, 26, 42–45], as we also observed in episode II of dog L11. *Anaplasma phagocytophilum* variant 4, which has sometimes been indicated as a “HGE variant” [22, 26], was detected in two episodes of dogs L11 and M12 and probably contributes to pathogenesis.

The higher prevalence rates for engorging (19.5%) than for questing ticks (2.5%) suggests that dogs play a role in delivering *A. phagocytophilum* to ticks, i.e. dogs are likely to have a reservoir function at a local scale [36, 46]. This canine reservoir function was demonstrated experimentally and suggested to have an enzootic function in public parks in Germany [18, 47–49]. In analogy with this, it might be that heavily tick infested dogs M12 and L11 established an enzootic cycle in their large garden by importing infected ticks from the woods where they were walked, thereby increasing the chance of enhancing a virulent canine clone. This hypothetical dynamic transmission by ticks between the dogs in this pack would have increased the chance of infection and re-infection analogous as in a flock of lambs [50].

The “Kempen” region where these dogs were living, is a known high-risk area for *A. phagocytophilum* infections [46, 51]. In humans, only a few cases have been described in this area [52, 53] but until now no associated clinical disease had been reported in dogs. By the longitudinal study of a pack of six Rhodesian ridgeback dogs we showed that *A. phagocytophilum* variant 4 infection was associated with typical disease in two of these dogs. Our results further indicate that re-infection and putatively persistence of *Anaplasma* infection occurs in naturally infected dogs. Confirmation may be obtained by detailed analysis, comparing the variable *msp2/p44* gene of amplified variants in the time line [54]. Comparing these variants to those causing known clinical cases in other regions is needed to determine the hazards to human health.

## Conclusions

In this study two lifelong cases of CGA are presented, showing that dogs can be naturally infected concurrently with *A. phagocytophilum* variant 1, variant 4 and the HGE agent. The ongoing presence of specific antibodies and *Anaplasma* DNA in one dog indicates one year of persisting infection. Treatment with doxycycline during recurring clinical episodes in the other dog resulted in transient clinical improvement and subsequent disappearance of specific antibodies and DNA suggesting that re-infection occurred.

## Additional file

Additional file 1: **Table S1.** Tabulated quarter year life events and serological, molecular, haematological and clinical data from six dogs, 1998 throughout 2009. Life histories of the six Rhodesian ridgeback dogs described per quarter year from 1998 through 2009. For each dog 8 columns were annotated: **a** Life events and surgical interventions, medications; **b** Rectal temperature taken in the veterinary clinic; **c** Clinical observations especially bleeding tendencies and immunological derived symptoms; **d** Other clinical observations as lameness, pain, fatigue, tumors; **e** Thrombocyte counts; **f** Erythrocyte counts or form observations; **g** White blood cell counts; **h** Serological and molecular (DNA) detection of infection. *Abbreviations*: N, neutrophilia; n, neutropenia; L, lymphocytosis; l, lymphopenia; M, monocytosis; m, monocytopenia; E, eosinophilia; e, eosinopenia; Morula, morulae detected in neutrophils; **P**/**P** both positive; **P**/N, serological positive/molecular negative; N/**P**, serological negative/molecular positive; N/N, both negative; -/N, -/**P**, N/-, **P**/-, -/-, either one or both not performed. *Note*: No abbreviation is given for normal cell count. (XLS 176 kb)
